# Identification of mitochondrial ATP synthase as the cellular target of Ru-polypyridyl-**β**-carboline complexes by affinity-based protein profiling

**DOI:** 10.1093/nsr/nwae234

**Published:** 2024-07-05

**Authors:** Wen-Jin Wang, Yu-Yi Ling, Yin Shi, Xiao-Wen Wu, Xuxian Su, Zheng-Qiu Li, Zong-Wan Mao, Cai-Ping Tan

**Affiliations:** MOE Key Laboratory of Bioinorganic and Synthetic Chemistry, School of Chemistry, Sun Yat-Sen University, Guangzhou 510006, China; MOE Key Laboratory of Bioinorganic and Synthetic Chemistry, School of Chemistry, Sun Yat-Sen University, Guangzhou 510006, China; Guangdong Basic Research Center of Excellence for Functional Molecular Engineering, Sun Yat-Sen University, Guangzhou 510006, China; School of Pharmacy, MOE Key Laboratory of Tumor Molecular Biology, Jinan University, Guangzhou 510632, China; MOE Key Laboratory of Bioinorganic and Synthetic Chemistry, School of Chemistry, Sun Yat-Sen University, Guangzhou 510006, China; MOE Key Laboratory of Bioinorganic and Synthetic Chemistry, School of Chemistry, Sun Yat-Sen University, Guangzhou 510006, China; School of Pharmacy, MOE Key Laboratory of Tumor Molecular Biology, Jinan University, Guangzhou 510632, China; MOE Key Laboratory of Bioinorganic and Synthetic Chemistry, School of Chemistry, Sun Yat-Sen University, Guangzhou 510006, China; Guangdong Basic Research Center of Excellence for Functional Molecular Engineering, Sun Yat-Sen University, Guangzhou 510006, China; MOE Key Laboratory of Bioinorganic and Synthetic Chemistry, School of Chemistry, Sun Yat-Sen University, Guangzhou 510006, China; Guangdong Basic Research Center of Excellence for Functional Molecular Engineering, Sun Yat-Sen University, Guangzhou 510006, China

**Keywords:** anticancer agents, bimolecular targets, chemical proteomics, ATPase inhibitor, ferroptosis

## Abstract

Ruthenium polypyridyl complexes are promising anticancer candidates, while their cellular targets have rarely been identified, which limits their clinical application. Herein, we design a series of Ru(II) polypyridyl complexes containing bioactive β-carboline derivatives as ligands for anticancer evaluation, among which **Ru5** shows suitable lipophilicity, high aqueous solubility, relatively high anticancer activity and cancer cell selectivity. The subsequent utilization of a photo-clickable probe, **Ru5a**, serves to validate the significance of ATP synthase as a crucial target for **Ru5** through photoaffinity-based protein profiling. **Ru5** accumulates in mitochondria, impairs mitochondrial functions and induces mitophagy and ferroptosis. Combined analysis of mitochondrial proteomics and RNA-sequencing shows that **Ru5** significantly downregulates the expression of the chloride channel protein, and influences genes related to ferroptosis and epithelial-to-mesenchymal transition. Finally, we prove that **Ru5** exhibits higher anticancer efficacy than cisplatin *in vivo*. We firstly identify the molecular targets of ruthenium polypyridyl complexes using a photo-click proteomic method coupled with a multiomics approach, which provides an innovative strategy to elucidate the anticancer mechanisms of metallo-anticancer candidates.

## Teaser

ATPase was verified as the molecular target of a ruthenium polypyridyl complex (**Ru5**) using photoaffinity-based protein profiling (PA-BPP) coupled with a multiomics approach. **Ru5** exhibits inhibitory effects on ATPase activity, leading to oxidative stress, mitophagy, ferroptosis and EMT suppression.

## INTRODUCTION

Although platinum-based anticancer therapeutics have been widely applied in clinics, they have drawbacks including toxic side effects and drug resistance [1–4]. Among non-platinum metallo-anticancer agents, ruthenium complexes are the most prominent, and four ruthenium complexes have successfully advanced to clinical trials [5,6]. In particular, Ru(II) polypyridyl complexes have attracted increasing attention due to their convenient structural modifications [7], alternative anticancer mechanisms different from those of platinum drugs [8], potential phototherapeutic applications [9,10], multifunctionalities integrating imaging and therapy [11,12], good biocompatibility and selectivity, etc. [13,14]. However, to the best of our knowledge, very little effort has been put into the cellular targets validation of Ru(II) polypyridyl complexes to date. Although complexes containing large planar ligands, such as dipyrido[3,2-a:2′,3′-c]phenazine (DPPZ) derivatives, are considered to target DNA, the prerequisite is that they must accumulate in mitochondria or nuclei to bind with DNA [15,16]. Moreover, the variation of the ligands shows a great impact on the anticancer properties of these complexes, which may be attributed to their different molecular targets in cells.

Activity-based protein profiling (ABPP) has been widely applied in medicinal chemistry and chemical/structural biology to identify drug targets, binding substrates or interaction sites [17–21]. Photoaffinity labeling (PAL) is a powerful tool to investigate non-covalent ligand–receptor interactions [22]. Typically, a covalent bond is formed between the photo-cross-linker and protein residues upon UV irradiation, which converts the interaction modes from reversible to irreversible binding [23–25]. Photoaffinity-based protein profiling (PA-BPP) has been widely used to identify the cellular targets of bioactive agents. Yao's group, as well as other groups, have put a lot of effort into developing highly efficient photo-cross-linkers with optimized stability and labeling specificity [26,27]. Among these linkers, the most interesting kind are the ‘minimalist’ bioorthogonal handle-containing photo-cross-linkers [27]. Without significantly affecting the structure and activity of the lead compounds, these linkers can improve labeling efficiency, and they are widely applied in the construction of irreversible inhibitors, proteome profiling, target identification, etc. [28,29].

β-Carboline alkaloids are a family of natural and synthetic products with a variety of biological activities [30]. They show potent anticancer activities through multiple mechanisms including intercalating into DNA [31], inhibiting topoisomerases I/II [32,33], impairing the cyclin-dependent kinases (CDKs) [34] and other oncoproteins [35,36]. Considering the easy coordination of nitrogen-containing heterocycles of β-carboline alkaloids with metal centers (e.g. ruthenium, iridium and copper), we and other groups have designed many metal-carboline complexes as anticancer agents in the past decade [37–40]. These complexes show interesting anticancer properties, including specific subcellular organelle localization and induction of different types of programmed cell death [41–43]. However, their molecular targets are still almost unknown, which is crucial for the understanding of their anticancer mechanisms and further applications.

**Scheme 1.**
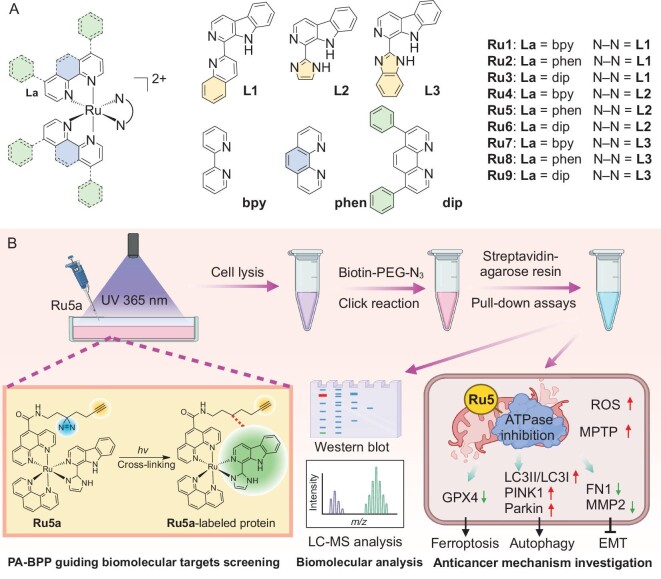
(A) The chemical structures of **Ru1**–**Ru9**. (B) Workflow for molecular target identification and anticancer mechanism investigation of **Ru5** using the photo-affinity probe **Ru5a** by PA-BPP.

In this work, nine Ru(II) polypyridyl complexes (Scheme 1A) containing β-carboline derivatives as ligands are synthesized, among which **Ru5** shows the highest anticancer activity. By using **Ru5a**, a structural analog of **Ru5** incorporating a photo-affinity tag, we identify that mitochondrial ATP synthase (ATPase) is the most important molecular target of **Ru5.** The findings from subsequent studies indicate that **Ru5** functions as an ATPase inhibitor, resulting in mitochondrial dysfunction and subsequently leading to ferroptosis and epithelial-to-mesenchymal transition (EMT) inhibition in cancer cells (Scheme 1B). The possible pathways through which **Ru5** interferes with mitochondrial function by inhibiting ATPase are elucidated by combined analysis of mitochondrial proteomics and RNA-sequencing. Moreover, **Ru5** can induce mitophagy and ferroptosis, and it also inhibits the EMT of cancer cells. Finally, *in vivo* evaluation shows that **Ru5** possesses a higher anticancer potency than cisplatin. Our study identifies the molecular targets of ruthenium polypyridyl complexes by integrating multiomics information, which gives insights into the action mechanisms of these potential metallo-anticancer candidates.

## RESULTS AND DISCUSSION

### Synthesis and structure–activity relationship study

Based on our previous work [43], we designed nine Ru(II) polypyridyl complexes by changing the auxiliary ligands (2,2'-bipyridine (bpy; **Ru1, Ru4** and **Ru7**); 1,10-phenanthroline (phen; **Ru2, Ru5** and **Ru8**); 4,7-diphenyl-1,10-phenanthroline (dip; **Ru3, Ru6** and **Ru9**)) to adjust the lipophilicity and the main β-carboline ligands (1-(quinolin-2-yl)-9H-pyrido[3,4-b]indole (**L1; Ru1**–**Ru3**); 1-(1H-imidazol-2-yl)-9H-pyrido[3,4-b]indole (**L2; Ru4**–**Ru6**); 1-(1H-benzo[d]imidazol-2-yl)-9H-pyrido[3,4-b]indole (**L3; Ru7**–**Ru9**)) to modify the binding ability with the targeted biomolecules. The β-carboline ligands are varied by keeping the core structure and introducing different pharmacophores (quinoline, imidazole and benzimidazole) to the C1-position. **L1**–**L3** were obtained by reacting tryptamine with the corresponding aldehyde in dry anisole as we previously reported [44,45]. **Ru1**–**Ru9** were synthesized by refluxing 1 equiv of the β-carboline ligand and the corresponding precursor *cis*-[Ru(N–N)_2_Cl_2_] followed by anion exchange with NH_4_PF_6_. The complexes were characterized by ^1^H nuclear magnetic resonance (NMR), ^13^C NMR spectroscopy, electrospray ionization mass spectrometry (ESI-MS) and elemental analysis (Supplementary Data, Figs S1–S34). X-ray diffraction shows that **Ru5** adopts a typical octahedral configuration (Fig. S35, Tables S1 and S2).

Subsequently, the lipophilicity and aqueous solubility of **Ru1**–**Ru9** were investigated. The results indicate that the Log*P*_o/w_ (oil-water partition coefficient) values of **Ru1**–**Ru9** range between −2.01 and 3.59 (Table S3). **Ru3, Ru5** and **Ru7** exhibit suitable Log*P*_o/w_ that can ensure good solubility and bioavailability [46]. By evaluating the linear relationship between concentration and absorbance, we found that the solubility of **Ru1**–**Ru9** in phosphate buffered saline (PBS) (with 1% dimethyl sulfoxide (DMSO)) was almost positively correlated with their lipophilicity. A linear relationship between the absorbance and the concentration is obtained for **Ru1, Ru2, Ru4, Ru5, Ru7** and **Ru8** up to at least 100 μM. The highest solubility of **Ru3, Ru6** and **Ru9** with dip as auxiliary ligands is lower than 40 μM under the same conditions (Fig. S36).

The antiproliferative activities of **Ru1**–**Ru9** were first evaluated on human cell lines including cervical carcinoma (HeLa), lung adenocarcinoma (A549), cisplatin-resistant A549R, prostate cancer (PC3), triple negative breast cancer (MDA-MB-231), malignant glioma (U87) and human lung fibroblast-like (HLF) cells (Table 1 and Table S4). The complexes containing β-carboline ligands with imidazole (**Ru4**–**Ru6**) and benzimidazole (**Ru7**–**Ru9**) as substituents have higher activity than those with quinoline (**Ru1**–**Ru3**) as substituents. **Ru3**–**Ru9** display relatively high anticancer activities towards the cancer cell lines tested and maintain good activity against cisplatin-resistant A549R cells. **Ru3**–**Ru9** also show a certain selectivity for cancer cells over normal cells, and **Ru2, Ru3, Ru5** and **Ru9** show higher selectivity index (SI) values than the other complexes.

**Table 1.** Half maximal inhibitory concentration (IC_50_, μM, 72 h) values of tested compounds towards different cell lines.^a^


Complexes	HeLa	A549	A549R	PC3	MDA-MB-231	U87	HLF	SI^b^

**Ru5**	3.1 ± 0.7	2.1 ± 0.8	3.3 ± 0.7	2.7 ± 0.4	7.7 ± 0.2	14.7 ± 0.7	33.9 ± 0.2	16.1
**Ru5a**	3.8 ± 0.1	2.5 ± 0.4	4.2 ± 0.6	3.4 ± 0.6	8.4 ± 0.6	13.4 ± 0.4	23.2 ± 0.3	9.3
Cisplatin	10.3 ± 1.3	5.9 ± 1.0	28.1 ± 4.2	13.1 ± 2.7	9.0 ± 0.2	14.8 ± 1.2	15.4 ± 1.1	2.6

a Cells were incubated with the compounds for 72 h. Data are presented as the means ± standard deviations (SDs). ^b^The SI (selectivity index) was calculated using the formula: SI = (IC_50_ for HLF)/(IC_50_ for A549).

Overall, the anticancer activities and cellular uptake efficacy (Fig. S37) of **Ru1**–**Ru9** are not completely positively related to their lipophilicity, represented as Log*P*_o/w_ (Table S3). Compared with the other complexes, **Ru5** shows better water solubility, a higher cellular uptake level and relatively higher antiproliferative activity, which may be due to its suitable lipophilicity and its capability to form hydrogen bonds with the targeted molecule through the imidazole ring. **Ru5** also shows a better selectivity for cancer cells, and its SI for cancerous A549 over normal HLF cells is 16.1 (Table 1 and Table S4), which makes **Ru5** a potential candidate for further investigation.

### Verification of ATP5F1 as the molecular target of Ru5 by PA-BPP

PA-BPP was then used to confirm the molecular targets of **Ru5** by modifying it with a ‘minimalist’ biorthogonal handle-containing photo-cross-linker. Through our structure–activity relationship analysis and comparison with other ruthenium complexes utilizing phen as the auxiliary ligands with similar coordination structures (using cisplatin as a control) [43,47,48], we propose that the β-carboline ligands play an important role in antiproliferative activity. To maintain the binding characteristics of the β-carboline moiety with the targeted biomolecules, we linked the photo-cross-linker to one of the auxiliary ligands of **Ru5**. Methylthiazolyldiphenyl-tetrazolium bromide (MTT) assay proves that **Ru5a** possesses similar antiproliferative activity to **Ru5** (Table 1).

**Figure 1.**
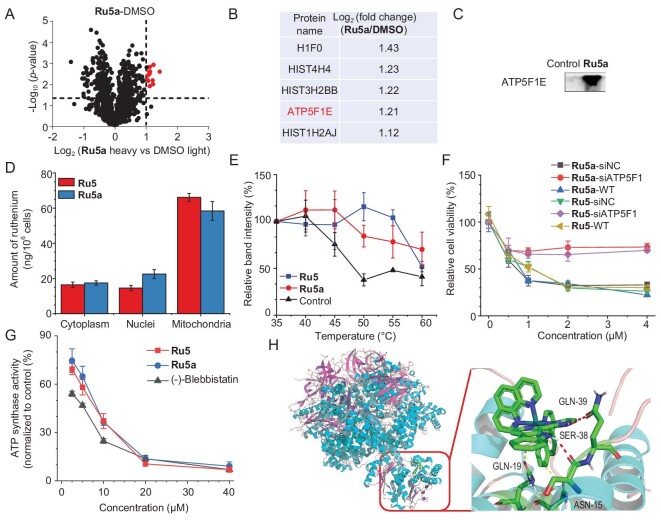
Validation of ATP5F1 as the molecular target of **Ru5**. (A) Proteins pulled down by streptavidin from A549 cells treated with **Ru5a** followed by PAL. The cells were treated with **Ru5a** (50 μM) for 12 h and irradiated with UV light for 20 min. (B) The top five peptides enriched by **Ru5a**-mediated PAL were identified with the assistance of SILAC-based chemoproteomic studies. H1F0: H1 histone family member 0; HIST4H4: histone cluster 4 H4; HIST3H2BB: histone cluster 3 H2B family member b; ATP5F1E: ATP synthase F1 subunit epsilon; HIST1H2AJ: histone cluster 1 H2A family member J. (C) Pull-down western blotting analysis of ATPase in A549 cells treated with **Ru5a**. (D) Cellular Ru content in cells treated with **Ru5**/**Ru5a** (20 μM, 12 h) measured by ICP-MS. (E) Impact of **Ru5**/**Ru5a** (10 μM) on the thermal stability of ATP5F1 measured by CETSA. (F) Impact on the anticancer activities of **Ru5**/**Ru5a** against wild type and ATPase knocking down (by siRNA) A549 cells. The relative cellular viability was calculated using the cells without drug treatment in each group as the control group. (G) Inhibition of **Ru5**/**Ru5a** on the enzymatic activity of ATPase. (-)-blebbistatin was used as the positive control. (H) Molecular docking of **Ru5** with ATPase (PDB: 3ZIA). The red dashes indicate the hydrogen bonds formed between **Ru5** and the amino acid residues (SER38 and GLN39). The yellow dashes indicate the hydrophobic interaction between **Ru5** and the amino acid residues (ASN15 and GLN19). Data are presented as means ± SD from three independent experiments.

Cell lysates of **Ru5a**-treated A549 cells were clicked onto the azide-conjugated biotin by UV irradiation, and the targeted proteins were enriched using the streptavidin-modified agarose resin. The samples treated with vehicle (1% DMSO) were used as controls. In A549 cells pretreated with **Ru5** (a competitor), the Stable Isotope Labeling with Amino Acids in Cell Culture (SILAC)-based chemo-proteomic pull-down assay for **Ru5a** reveals no significant protein enrichment (Fig. S38), which indicates that **Ru5** and **Ru5a** share the same cellular targets. The peptides after trypsin digestion were isotopically labeled by reductive demethylation for liquid chromatography-mass spectrometry (LC-MS)/MS analysis. A total of 816 proteins are detected from **Ru5a**-treated and control samples, among which 13 proteins (Fig. 1A, Table S5) are differentially enriched in **Ru5a**-treated samples (*P* ≤ 0.05, log_2_(fold change) ≥ 1).

The top five enriched peptides include H1F0 (H1.0 linker histone), HIST4H4 (histone cluster 4), HIST3H2BB (histone cluster 3 H2B family member b), ATP5F1E (ATP synthase F1 subunit epsilon) and HIST1H2AJ (H2A clustered histone 14; Fig. 1B). ATP5F1E belongs to the F1 subunit of ATPase, a protein mainly localized in mitochondria [49]. The other four peptides are components of histone in nuclei, which may be due to the relatively high expression levels of these proteins leading to a high background enrichment signal. Moreover, another peptide (ATP5F1A, ATP synthase F1 subunit alpha) in the F1 subunit of ATPase is also differentially enriched in **Ru5a**-treated samples. The pull-down assays followed by immunoblotting show that **Ru5a** can efficiently pull down ATP5F1 (Fig. 1C). Inductively coupled plasma-mass spectrometry (ICP-MS) experiments show that **Ru5**/**Ru5a** are mainly located in mitochondria (Fig. 1D). We propose that **Ru5** may bind to ATPase in cells. The cellular thermal shift assay (CETSA) also proves that the thermal stability of ATP5F1 is increased upon **Ru5**/**Ru5a** treatment (Fig. 1E and Fig. S39). Moreover, compared to the wild-type cells, siRNA-mediated knockdown of ATPase alleviates the anticancer effects of **Ru5** and **Ru5a** (Fig. 1F), while ATPase knockdown using siRNA has no significant impact on A549 cell proliferation (Fig. S40).

Both **Ru5** and **Ru5a** display a dose-dependent inhibition on the enzymatic activity of ATPase *in vitro* (Fig. 1G), and the inhibition constant (*K*i) measured for **Ru5** (6.2 ± 1.4 μM) and **Ru5a** (8.1 ± 1.5 μM) is comparable to that of the positive control (-)-blebbistatin (3.3 ± 0.5 μM). The estimated inhibition constant (*K*i = 9.22 μM; Table S6) is close to the *K*i value obtained from the enzymatic inhibition assay.

Molecular docking using the crystal structures of ATP synthase F1 (PDB: 3ZIA) and **Ru5** shows that **Ru5** binds to the interspace between the γ and ε subunit, and two hydrogen atoms on the indole and imidazole rings of the β-carboline ligand of **Ru5** can form hydrogen-bonds (red dashes) with the residues SER38 and GLN39 of ATPase F1 ε subunit, respectively (Fig. 1H). Moreover, the benzene ring of the β-carboline ligand of **Ru5** can form hydrophobic interaction (yellow dashes) with ASN15/GLN19 on ATPase F1 ε subunit. The roles of SER38 and GLU39 were further validated by substituting them with compact-sized glycine, hydrophobic alanine or steric phenylalanine. The results demonstrate that mutations in either SER38 or GLU39 lead to disruption of the hydrogen bonds between **Ru5** (Fig. S41) and ATPase and result in a decrease in the binding affinity between **Ru5** and ATPase (Table S6).

The docking results of the other complexes with ATPase indicate that **Ru1, Ru2, Ru3, Ru4** and **Ru7** cannot form hydrogen bonds with the protein (Fig. S42). **Ru8** and **Ru9** can each form a hydrogen bond with residues GLU179 and ASP169 via the indole imine group on L3, respectively. **Ru6** can form two hydrogen bonds with residue THR40 via the indole and imidazole imine groups on L2. The calculation of the binding energy shows that **Ru5** possesses the most favorable affinity towards ATPase (Table S7). Based on these results, we infer that ATP synthase is an important cellular target of **Ru5**, and the β-carboline ligand plays an important role in target binding.

### Ru5 induces mitochondrial dysfunction

**Figure 2.**
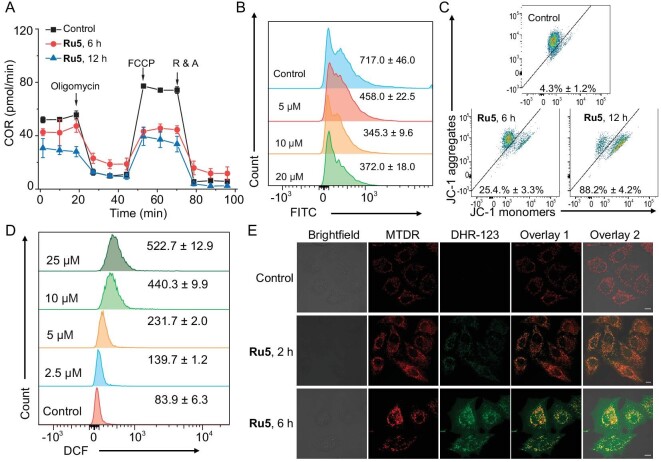
Ru5 causes mitochondrial dysfunction. (A) Impact of **Ru5** (5 μM) on mitochondrial respiration of A549 cells measured by Seahorse. The OCR was measured under basal conditions, and after the sequential addition of oligomycin (1 μM), carbonyl cyanide 4-(trifluoromethoxy)phenylhydrazone (FCCP, 0.8 μM) and a mixture of rotenone (0.5 μM) and antimycin A (0.5 μM, R&A). Data are presented as means ± SD from three independent experiments. (B) The impact of **Ru5** on MPTP. The cells were treated with **Ru5** for 6 h and analyzed by flow cytometry. *λ*_ex_ = 488 nm, *λ*_em_ = 520 ± 20 nm. (C) Impact of **Ru5** (5 μM) on MMP measured by JC-1 staining. The cells were treated with **Ru5** for 6 h or 12 h, stained with JC-1 for 10 min, and analyzed by flow cytometry. *λ*_ex_ = 488 nm, *λ*_em_ = 525 ± 30 nm (JC-1 monomers) or 585 ± 30 nm (JC-1 aggregates). (D) Effect of **Ru5** on cellular ROS level. The cells were stained with 2',7'-dichlorodihydrofluorescein diacetate (DCFH-DA) and treated with **Ru5** (5 μM). DCF: *λ*_ex_ = 488 nm, *λ*_em_ = 520 ± 30 nm. (E) Impact of **Ru5** on mitochondrial ROS generation. A549 cells were treated with **Ru5** (10 μM) and stained with DHR-123 (5 μM) and MTDR (100 nM). **Ru5**: *λ*_ex_ = 488 nm; *λ*_em_ = 600 ± 20 nm. DHR-123: *λ*_ex_ = 514 nm; *λ*_em_ = 540 ± 20 nm. MTDR: *λ*_ex_ = 633 nm; *λ*_em_ = 700 ± 20 nm. Scale bar: 20 μm.

As **Ru5** is located in mitochondria and inhibits ATPase related to mitochondrial respiration, we then evaluated its impact on oxygen consumption rate (OCR) using the Seahorse XF analyzer. **Ru5** inhibits ATP-linked respiration, basal respiration and maximal respiration capacity, and it also increases the proton leak and alters non-mitochondrial respiration (Fig. 2A and Fig. S43). Accordingly, **Ru5** treatment leads to an increase in the opening of the mitochondrial permeability transition pore (MPTP) of A549 in a concentration-dependent manner (Fig. 2B). 5,5′,6,6′-tetrachloro-1,1′,3,3′-tetraethylbenzimidazolyl-carbocyanine iodide (JC-1) staining assay confirms the loss of MMP (mitochondrial membrane potential) in **Ru5**-treated A549 cells (Fig. 2C). Moreover, **Ru5** elevates reactive oxygen species (ROS) levels dose-dependently in cells (Fig. 2D). The colocalization of the fluorescence of dihydrorhodamine 123 (DHR-123) with MitoTracker Deep Red (MTDR) indicates ROS are mainly generated in mitochondria (Fig. 2E).

### Combined analysis of mitochondrial proteomics and RNA-sequencing

**Figure 3.**
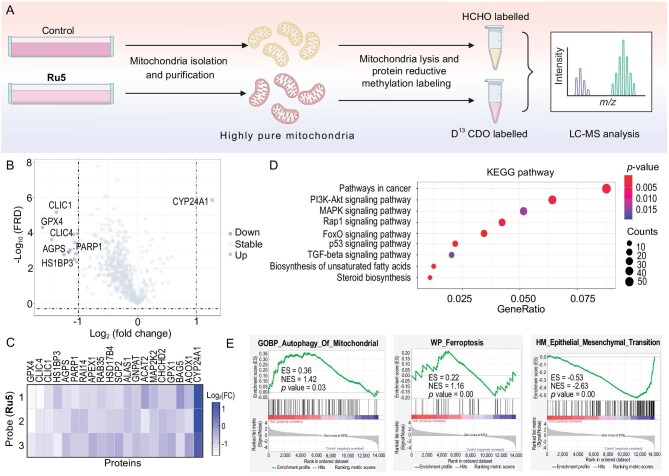
Impact of **Ru5** on mitochondrial proteomics and whole-cell transcriptome. (A) Schematic overview of the workflow of mitochondrial proteomics. (B) The volcano plot shows the DAPs in the mitochondria of A549 cells treated with **Ru5** (10 μM, 12 h) compared with the control samples. (C) The relative expression of differentially expressed proteins (DEPs) identified by mitochondrial proteomic analysis. Data are presented as means ± SD from three independent experiments. (D) KEGG analysis of DEGs identified by RNA-seq of A549 cells after **Ru5** (10 μM, 12 h) treatment. (E) GSEA analysis of the DEG genes in **Ru5**-treated groups in the mitophagy pathway, ferroptosis and epithelial-mesenchymal transition pathway.

Given **Ru5** is mainly localized in mitochondria as an ATPase inhibitor, mitochondrial proteomics is used to further investigate its action mechanisms (Fig. 3A). Of the 556 proteins identified (Supplementary Data S1), 20 proteins (up-regulated: 1; down-regulated: 19) are found to be differentially accumulated proteins (DAPs; |fold change| ≥ 1.8, false discovery rate (FDR) ≤ 0.01; Fig. 3B and Table S8). These DAPs are mainly involved in ion channels, lipid oxidation and intracellular redox balance. The top five down-regulated proteins are GPX4 (glutathione peroxidase 4), CLIC4 (chloride intracellular channel 4), CLIC1 (chloride intracellular channel 1), HS1BP3 (heterochromatin protein 1 binding protein 3) and AGPS (alkylglycerone phosphate synthase, Fig. 3C). CLIC4 and CLIC1 are intracellular chloride channel proteins mediating Cl^−^ influx through 2Cl^−^/H^+^ exchange [50]. As the counterion, Cl^−^ provides the electrical shunt for proton pumping by ATPase [51]. Therefore, the decreased accumulation of CLIC4 and CLIC1 may be caused by ATPase inhibition [52]. Interestingly, GPX4, GPX1 and APEX1 are antioxidant selenoenzymes involved in ferroptosis, especially for GPX4 [53]. ALAS1 is involved in heme synthesis, and ALAS1 inhibition can impair iron utilization, resulting in iron overload and ferroptosis [54]. Intriguingly, AGPS (lipid biosynthesis), HSD17B4 (peroxisomal beta-oxidation pathway for fatty acids), GNPAT (lipid biosynthesis and metabolism), ACAT2 (lipid metabolism) and ACOX1 (fatty acid beta-oxidation) are all related to lipid metabolism/oxidation, which is consistent with the fact that ferroptosis is driven by lipid peroxidation [55]. HS1BP3 (formation of LC3-positive autophagosomes), Rab35 (membrane trafficking) and BAG5 (mitophagy regulating) are involved in the regulation of autophagy [56–58]. The reported functions of MAP2K2 (energetic metabolism) [59], CHCHD2 (hypoxia) [60] and PARP1 (DNA repair) [61] are all closely associated with mitochondrial functionalities. Finally, the only up-regulated protein, CYP24A1, is a cytochrome P450 oxidoreductase, which is reported to influence phospholipid peroxidation during ferroptosis [62].

RNA-seq was further performed to clarify the antitumor mechanisms of **Ru5**. An average of 95.96% mappability and 46.1 million qualified fragments for each RNA-seq sample are obtained (Table S9). High correlations (*R* > 0.95, Fig. S44) representing the reproducibility are obtained for parallel samples. As compared with the control samples, the total number of DEGs (differential expression genes; |Fold change| ≥ 2.0; FDR ≤ 0.05; Supplementary Data S2) for **Ru5**-treated samples is 1517 (up-regulated: 673; down-regulated: 844). GO (gene ontology) term analysis shows that **Ru5** treatment influences the cellular process, cell part and binding (Fig. S45). KEGG (Kyoto Encyclopedia of Genes and Genomes) analysis shows that **Ru5** influences pathways including PI3K-AKT (phosphoinositide 3-kinase and AKT), MAPK (mitogen-activated protein kinase), Rap1 (Ras-association proximate 1), FoxO (forkhead box O), p53 (tumor suppressor p53), TGF-β (transforming growth factor-β), biosynthesis of unsaturated fatty acids and steroid biosynthesis (Fig. 3D). Gene set enrichment analysis (GSEA) shows that cell death induced by **Ru5** is positively associated with autophagy of mitochondrion and ferroptosis. Besides, the GSEA analysis also shows the up-regulation of the mitophagy pathway and ferroptosis pathway in **Ru5**-treated cells (Fig. 3E). Moreover, gene signatures related to EMT are down-regulated in **Ru5**-treated cells (Fig. 3E). In all, these data show that **Ru5** can mainly influence pathways related to mitochondrial redox homeostasis, ferroptosis, mitophagy and EMT.

### Ru5 induces mitophagy and ferroptosis, and inhibits EMT *in vitro*

**Figure 4.**
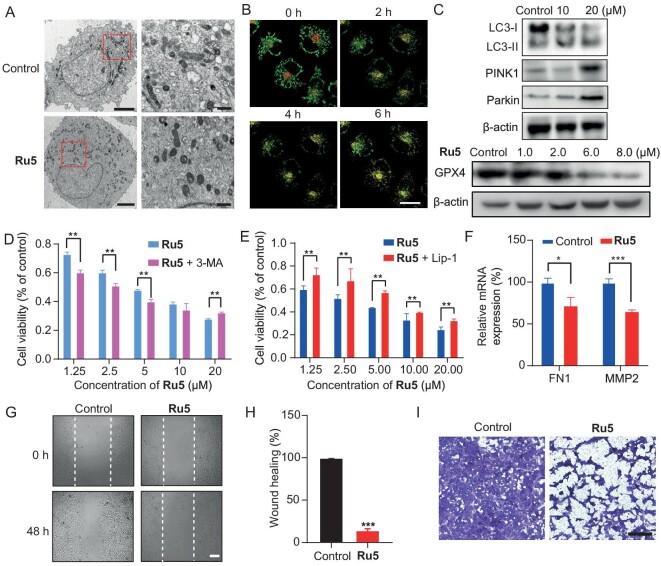
Ru5 induces mitophagy and ferroptosis and inhibits EMT *in vitro*. (A) TEM images of A549 cells treated with vehicle (1% DMSO) or **Ru5** (10 μM, 12 h). Red rectangles represent the region enlarged. Scale bar: 5 μm and 500 nm (enlarged). (B) The morphological examination of mitochondria in A549 cells treated with **Ru5** (10 μM) for the indicated time intervals. MTG: *λ*_ex_ = 514 nm; *λ*_em_ = 540 ± 20 nm. LTDR: *λ*_ex_ = 633 nm; *λ*_em_ = 700 ± 20 nm. Scale bar: 20 μm. (C) Western blotting analysis showing the impact of **Ru5** (24 h) on the expression of proteins related to mitochondrial dysfunction and ferroptosis. (D and E) The impact of different inhibitors on antiproliferative activity of **Ru5** (24 h). (F) RT-qPCR analysis of EMT-related genes in A549 cells after **Ru5** (10 μM, 12 h) treatment. (G) The impact of **Ru5** (5 μM) on the migration of A549 cells was measured by a wound healing assay. Scale bar: 100 μm. (H) Quantitative analysis of the impact of **Ru5** on wound healing. (I) Selective images of the impact of **Ru5** (5 μM, 48 h) on the migration of A549 cells detected by transwell assay. Scale bar: 100 μm. Data are presented as means ± SD from three independent experiments. *P ≤ 0.05, **P ≤ 0.01, ***P ≤ 0.001, by Student's t-test.

As shown by transmission electron microscopy (TEM) observation, in cells treated with **Ru5**, some mitochondria are enclosed by a bilayer membrane with increased density, and many of them are wrapped in double-layered membrane structures (Fig. 4A). At the same time, the morphology of the nucleus is unaffected. These features are typical morphological characteristics of autophagy and ferroptosis [63]. In A549 cells co-labeled with MitoTracker Green (MTG) and LysoTracker Deep Red (LTDR), **Ru5** treatment causes a gradual morphological alternation in mitochondria from fibrous to dot-like. Prolonged incubation leads to a time-dependent overlap of the emission of MTG and LTDR (Fig. 4B and Fig. S46), which confirms the occurrence of mitophagy. In cells treated with **Ru5**, western blotting shows the conversion of LC3I to LC3II, and an increased expression of PINK1 (PTEN-induced putative kinase 1) and Parkin, along with a decreased expression of GPX4 (Fig. 4C).

We then used different specific inhibitors to further confirm the modes of cell death induced by **Ru5**. These inhibitors act on autophagy (3-methyladenine, 3-MA, Fig. 4D) [64], ferroptosis (liproxstatin-1, Lip-1, Fig. 4E) [65], necroptosis (necrostatin-1, Nec-1, Fig. S47A) [66] and apoptosis (Z-VAD-FMK, Fig. S47B) [67]. The effect of 3-MA on the antiproliferative activity of **Ru5** is concentration-dependent, with lower and higher concentrations enhancing and inhibiting the antiproliferative activity of **Ru5**, respectively. Thus, **Ru5** induces cytoprotective autophagy at lower concentrations, while autophagic cell death is induced at higher concentrations. As expected, Lip-1 can relieve the antiproliferative activity of **Ru5**. However, Nec-1 and Z-VAD-FMK show no effect on the cell viability of **Ru5**-treated cells. All these results show that inhibition of ATPase by **Ru5** causes mitochondrial dysfunction and ROS accumulation, leading to mitophagy and ferroptosis.

Based on the GSEA and KEGG analysis, we then evaluate the impact of **Ru5** on EMT and angiogenesis. Firstly, real-time quantitative polymerase chain reaction (RT-qPCR) experiments (Fig. 4F) confirm that **Ru5** can down-regulate genes including CDH1 (cadherin 1) [68], FN1 (fibronectin 1) [69] and MMP2 (matrix metallopeptidase 2) [70]. Wound healing assays (Fig. 4G and H) and transwell migration assays (Fig. 4I) verify that **Ru5** can markedly decrease the migration rates of the A549 cells. All these results indicate that **Ru5** can induce mitophagy and ferroptosis, and also simultaneously affects tumor invasive and metastatic processes including EMT and angiogenesis by inhibiting ATPase.

### Ru5 exhibits potent anticancer activities *in vivo*

**Figure 5.**
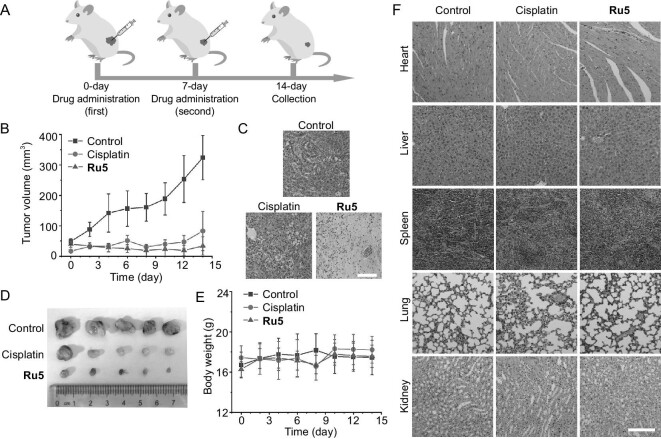
**Ru5** exhibits potent anticancer activities *in vivo*. (A) Schematic illustration of *in vivo* therapeutic protocol. (B) Graphs of tumor volumes of nude mice after treatment with **Ru5** (5 mg kg^−1^), cisplatin (5 mg kg^−1^) and PBS. The intratumoral injections were performed every 7 days. Data are presented as means ± SD, *n* = 5. (C) Immunohistochemical analysis of hematoxylin-eosin (H&E) staining in tumor sections from mice with different treatments. Scale bar: 50 μm. (D) Tumors separated from nude mice. (E) The average body weight of the mice in each group during the treatment process. Data are presented as means ± SD, *n* = 5. (F) H&E staining of the main organs from mice with different treatments. Scale bar: 100 μm.

Lastly, the *in vivo* anticancer activity of **Ru5** was assessed via a A549 xenograft nude mice model (Fig. 5A). Mice were treated with PBS, cisplatin (5 mg kg^−1^) or **Ru5** (5 mg kg^−1^) for 14 days, during which body weights and tumor volumes were monitored. **Ru5** showed better tumor inhibition than cisplatin and PBS (Fig. 5B–D; Fig. S48). Compared with the control groups, the tumor growth inhibitory rates of **Ru5** and cisplatin reached ∼89.6% and ∼74.4%, respectively. Moreover, no significant body weight loss (Fig. 5E) or organ damage (Fig. 5F) was observed during therapy for **Ru5**. All these results show that **Ru5** has great potential to be developed as an anticancer candidate.

## CONCLUSION

Metallo-anticancer agents have gained significant attention in recent years due to their potential to overcome chemotherapy resistance and target specific pathways in cancer cells. In this work, we report nine Ru(II) polypyridyl complexes containing β-carboline alkaloid derivatives as ligands, among which **Ru5** is identified as a potent anticancer candidate. PA-BPP confirms that mitochondrial ATPase is one of the most important targets of **Ru5**. Using the combined analysis of mitochondrial proteomics, RNA-seq and cell-based validations, we show that **Ru5** can induce mitochondrial dysfunction, mitophagy and ferroptosis. Moreover, **Ru5** can inhibit EMT and angiogenesis. The findings shed light on the complex interplay between mitochondrial function, EMT and ferroptosis in cancer cells. Finally, *in vivo* experiments demonstrate that **Ru5** possesses a better antitumor effect than cisplatin. In conclusion, we have identified the molecular targets of Ru(II) polypyridyl complexes by coupling PAL with a multiomics approach, which provides an innovative strategy to elucidate the anticancer mechanisms of metallo-anticancer agents.

## METHODS

Detailed information about the materials and experimental procedures can be found in the supplementary data. All animal operations followed the guidelines of the Sun Yat-Sen University Animal Care and Use Committee.

## Supplementary Material

nwae234_Supplemental_Files
